# Current Competencies of Game Facilitators and Their Potential Optimization in Higher Education: Multimethod Study

**DOI:** 10.2196/25481

**Published:** 2021-05-05

**Authors:** Jannicke Baalsrud Hauge, Heinrich Söbke, Thomas Bröker, Theodore Lim, Angelo Marco Luccini, Maksims Kornevs, Sebastiaan Meijer

**Affiliations:** 1 KTH Royal Institute of Technology Södertalje Sweden; 2 BIBA – Bremer Institut für Produktion und Logistik GmbH Bremen Germany; 3 Bauhaus-Universität Weimar Weimar Germany; 4 Nuremberg Institute of Technology Nuremberg Germany; 5 Heriot Watt University Edinburgh United Kingdom; 6 Succubus Interactive Nantes France; 7 KTH Royal Institute of Technology Stockholm Sweden

**Keywords:** facilitation, higher education, competency, simulation, gaming, educational games

## Abstract

**Background:**

Serious games can be a powerful learning tool in higher education. However, the literature indicates that the learning outcome in a serious game depends on the facilitators’ competencies. Although professional facilitators in commercial game-based training have undergone specific instruction, facilitators in higher education cannot rely on such formal instruction, as game facilitation is only an occasional part of their teaching activities.

**Objective:**

This study aimed to address the actual competencies of occasional game facilitators and their perceived competency deficits.

**Methods:**

Having many years of experience as professional and occasional facilitators, we (n=7) defined requirements for the occasional game facilitator using individual reflection and focus discussion. Based on these results, guided interviews were conducted with additional occasional game facilitators (n=4) to check and extend the requirements. Finally, a group of occasional game facilitators (n=30) answered an online questionnaire based on the results of the requirement analysis and existing competency models.

**Results:**

Our review produced the following questions: Which competencies are needed by facilitators and what are their training needs? What do current training courses for occasional game facilitators in higher education look like? How do the competencies of occasional game facilitators differ from other competencies required in higher education? The key findings of our analysis are that a mix of managerial and technical competencies is required for facilitating serious games in higher educational contexts. Further, there is a limited or no general competence model for game facilitators, and casual game facilitators rarely undergo any specific, formal training.

**Conclusions:**

The results identified the competencies that game facilitators require and a demand for specific formal training. Thus, the study contributes to the further development of a competency model for game facilitators and enhances the efficiency of serious games.

## Introduction

Games in education offer alternative means to balance the learning of a subject, along with its absorption, and application. Games used for nonentertainment purposes are often termed *serious games* [1-3]. However, there are also several other definitions and terms, including *simulation games*, *educational games*, or *digital educational games* [2], with the games being be single player or multiplayer games. Furthermore, the usage in an educational setting differs, but within engineering education, these are often facilitated and based on experiential learning models. For instance, it might be assumed that a gameplay cycle reflects the 4 phases of Kolb’s experiential learning cycle [4]: a recursive cycle of experiencing, reflecting, thinking, and acting to help learners increase their learning power.

Experiential learning, as well as gameplay, requires learners to take responsibility for their own learning. However, teachers should not overestimate the students’ level of achievement in conceptual understanding [5]. A particular experience of an individual is often idiosyncratic to their perception of that experience and outside the control of the teacher. This dichotomy could encourage teachers to provide answers to students. However, the key is to avoid answers and instead develop the learners’ capability to find answers on their own [4,6-8].

Purposeful learning processes based on serious games are often known as game-based learning (GBL). The learning outcomes supported with GBL are diverse. For example, meta-skills can be trained in simulation games [9,10], affective learning outcomes are achieved [11], engagement of the learner is increased [12], system interdependencies are demonstrated [13,14], and pure factual knowledge is taught [15,16], all based on GBL. De Gloria et al [17] distinguish between factual knowledge and skills as learning outcomes supported by GBL. Meanwhile, Egenfeldt-Nielsen [18] discusses a wide range of GBL scenarios and the learning outcomes achieved. In a systematic review on the use of serious games, Boyle et al [19] identify the categories of science, technology, engineering and mathematics; health; business and economics; and languages as domains for GBL. From the fundamental perspective of game studies, Klabbers [20] discusses the issues of knowledge building in games, while Shaffer has coined the term *epistemic games* [21]. Games can be seen as environments in which experiences are gained, which are processed by the learners, thus leading to learning processes. The theory of situated learning [22] is one of the approaches to understanding the nature of GBL: games may be seen as a context in which experiences may be gained. Another approach is that of sense-making [23]: the experiences generated by games must be interpreted and opened up by the learners themselves. Without an emphasis on any of these and other approaches, it becomes obvious that GBL poses different demands on the teacher compared to traditional teaching concepts, such as traditional classroom settings. Accordingly, the role of a teacher in GBL must differ from that of a traditional classroom setting. Here, the teacher as a facilitator can improve the permeance of learning by activating the affordances of GBL. Chapman et al [24] have outlined several characteristics to define experiential learning through games. Of these, the mixture of content and process (a balance between experiential activities and the underlying content or theory), the role of reflection (students gaining insight into themselves and their interactions), and meaningful relationships (getting students to see their learning in the context of the entire world) have direct contextual alignment to GBL. If properly facilitated, game-based learning has the potential to allow students to experience all 4 phases of Kolb's experiential learning cycle.

Facilitation should not be mistaken as a resource bank to help develop students’ confidence in the learning process. However, facilitators must set the right ambience where students feel engaged, valued, trusted, and respected [25]. They must create a space where students can express different viewpoints without a fear of saying the wrong thing, they must be sensitive to cultural experiences, and they must adapt the pace of learning while ensuring the learners understand and absorb the subject [24]. Students need to understand why they are doing something; otherwise, they may not reach the intended learning outcomes.

This paper asks where and what facilitation competency is necessary and to what extent competency models have been applied or administered by those who engage or use games in education. This kind of implementation (of facilitation competency and of competency model) might be somewhere in between facilitating collaborative knowledge building, and being aware of patterns in students’ learning behavior (as an individual and in a group) towards problem-solving. To better understand this relation, the mode of research could be reshaped to question the role of reflection with respect to the content through a facilitator's ability to recognize students’ knowledge acquisition under guided and unguided independent learning; to apply knowledge building mechanisms into the GBL process; and to differentiate between GBL and gamified learning, and other blended learning (including conventional) approaches. The overarching research examines a facilitator’s familiarity with competency models in GBL and whether this knowledge has any impact on pedagogy.

Consequently, an attempt has been made to assimilate the external and internal factors that are relevant to GBL, including the experience of the facilitator, the types of games used, the structure of the course, the level and size of cohorts, the equipment and technology used, and the environment. The autoethnography approach taken draws from our experiences and combines this with an advocacy and participatory approach aimed at understanding and acquiring new knowledge regarding the facilitation of GBL. The primary purpose of our study is to impart practical actions for future course design. What follows is a quantitative analysis to identify facilitator roles and to describe characteristics of facilitation (including models) that may be associated with successful GBL.

Our objective was to provide insights into the competency models for facilitators before conducting a self-observation and reflexive investigation into facilitating GBL. We then present and discuss the qualitative and quantitative analysis before concluding with some remarks on the facilitation process and associated competency models for GBL.

## Methods

Our study attempted to assimilate the external and internal factors that apply to game facilitation, including the experience of the facilitator, the types of games used, the structure of the course, the level and size of cohorts, the equipment used, and the environment. Using an autoethnographic and participatory approach, we drew from our experiences to understand and acquire new knowledge on facilitating GBL, with a primary purpose to impart practical actions for future course design. A quantitative analysis followed to identify facilitator roles and to describe characteristics of facilitation (including models) that may be associated with successful GBL. The research questions to be answered were the following:

Which competencies are particularly needed by facilitators, and what are the training requirements for facilitators? What do current training courses for occasional game facilitators in higher education look like? How do the competencies of occasional game facilitators differ from other competencies required in higher education?

The study offers perspectives into competency models for facilitators from the literature (see Findings From the Literature Review subsection). We then conducted a self-observation and reflexive investigation on facilitating GBL (n=7; see Authors’ Experience Review subsection). The results were complemented by guided interviews with game facilitators from the field of engineering education (n=4; see External Experience Review subsection). The outcomes were compiled into a questionnaire with closed and open questions. The closed questions included an assessment of existing competency models for game facilitation–related activities. The questions determined the degree to which the competencies included in the models were considered important for game facilitation. Open questions aimed at contributing to a supplementation of the competency models. The questionnaire was distributed via social media. The qualitative and quantitative analyses of the answers (N=30) are presented (Questionnaire subsection) and discussed (see Discussion section) in this paper. We conclude with remarks on the facilitation process and associated competency models for GBL (see Summary and Future Work subsection).

## Results

### Findings From Literature Review

Competencies and their models have superseded the long-established job analysis and their resulting task descriptions in human resource management. For today’s fast-changing world and its complex situations, job analysis turned out to be an inefficient methodology [26]. Competency models define a structured collection of competencies that organizations can align to their objectives and strategies [27]. They support organizations to handle current and future situations [28] and *“*are used to hire, train, evaluate and promote employees on the same attributes*”* [27]. The primary perception of competency as the ability to deal with typical situations (as discussed by Mudra [29]) has meanwhile grown into the ability to deal with complex situations [28,30]. Boulter et al [31], cited in [32], suggested a process of 6 stages to define competency models: defining performance criteria, choosing a sample of people, collecting data, analyzing the data, validating the found results, and applying the model in practice.

From the rising complexity of situations and problems, the role of the facilitator has emerged to support organizations in the process of transition and transformation [33]. Expert consultants diagnose problems and prescribe a therapy, while facilitators are process consultants. Facilitators organize and release information in a methodical and interactive manner instead of focusing on the group’s output [34]. In this way, they improve a group’s effectiveness [35]: *“*the facilitator supports and guides, reassures and encourages […] and never ‘teaches’ the meaning of what is happening*”* [25].

Nelson and McFadzean [34] developed a conceptual competency model for facilitators and defined 7 of competencies: understanding context; technical, rational, interpersonal, task, and human process competencies; and other personal characteristics. These authors further differentiated these competencies into 3 levels, ranging from an initial to an expert facilitator. Stewart [33] generated a facilitator competency model from a qualitative study of groups in facilitation contexts and validated it through a survey. It comprises 5 areas of competencies: interpersonal competency (as communication and further skills), management process competency, knowledge competency, and personal characteristics.

In higher education, the role of a facilitator is just one role of several alternatives for teaching. In a literature review, Hoidn and Kärkkäinen [36] summarized instructional effectiveness and process–outcome research on competencies for effective higher education teaching. They concluded that effective teachers start their series of lessons with direct instruction and decrease control with the growing mastery of the students *“*to allow for independent and fluent performance by the students themselves”. However, they conclude that an ultimate consensus on effective teaching is difficult because of the manifold contexts of teaching and identify enthusiasm as a key element of instructional effectiveness. Blašková et al [37] proposed a competency model for university teachers based on theoretical analysis and a survey among students. The competency model they proposed comprises professional, educational, motivational, communicational, personal, science and research, and publication competency. Each competency is connected to indicators of positive and negative behavior. Both Blašková et al [37] and Hoidn and Kärkkäinen [36] emphasize the role of motivational competency.

In a similar concept involving the range between controlled lessons and independent performance of students, Leigh and Spindler [25] adopted the idea of closed and open games to develop competencies of simulation and game facilitators. The facilitators’ competencies range according to their degree of emotional detachment and the number of possible learning goals they allow. An observation from this study indicated that a facilitator’s preference on open or closed games was a product of learning preferences and personality type. Kortmann and Peters [38] proposed a competency model for game facilitation, identifying the differences between facilitating a simulation game and leading generic groups. They point out that the facilitation of games should require more competencies, as games can serve more aims. By interviews with a group of facilitation experts, they validated the model. However, the authors expressed concerns that competencies at a subconscious level might have been missed.

Kortmann and Peters [38] identified similar competencies between facilitating game and leading generic group sessions, but they also indicated some dissimilarities, suggesting that game facilitators may require additional competencies. Taken together, the literature suggests that participants of games play within a model that requires game facilitators to have additional abilities. Game facilitators have to be aware that games are a means of learning about real-life situations and thus need to switch and adapt quickly to different roles and styles during gameplay. To facilitate a session and delegate control to the game environment, they have to comprehend the game as an immersive instrument and understand its elements and mechanics. Finally, facilitators have to translate between the game environment and reality so that the participants transfer their experiences to the real world and back [38].

### Authors’ Experience Review

This section synthesizes our experiences as game facilitators (the author group). We reflected individually and independently upon a set of questions organized in 3 blocks: (1) experience with game facilitation, (2) knowledge on competency models, and (3) required competencies and the biggest discrepancy perceived between required and present competency.

The following information was collected to understand our experience with game facilitation: types of games used, number of games used, description of the educational settings for using the games, reasons for using games in teaching, changes in our perspective towards teaching, changes of our attitudes towards teaching, changes over time in the facilitation, and if we applied GBL in different settings.

The analysis shows that the group had extensive experience with facilitation. All but one participant had facilitated several games, a few had done so for decades, and all had done so for at least 4 years. Most of the group facilitated games within engineering or management studies at undergraduate and postgraduate level. Both fields have a strong tradition of using experiential learning [39,40]. In addition, 5 of the participants also facilitated games on an executive and vocational level.

We found that our group had played a large variety of games, had designed and developed games, and had used games developed by others. The games comprised multiplayer games, and both board or haptic games and digital games. The topics addressed in the games could be divided into managerial competency development and engineering topics, like bridge construction, sustainable manufacturing, and product development. Specific games addressing well-known problems, such as the bullwhip effect and capacity constraints (ie, where the player learns about consequences of a decision and strategies for solving a problem) were applied, as well as games addressing communication, cooperation, and team skills. A difference between graduate courses and undergraduate courses became apparent. At the undergraduate level, the focus is on factual and procedural knowledge reflection. At the master level, it is on developing new knowledge and higher-order thinking. This is in line with how universities structure their undergraduate and postgraduate studies and with current taxonomies of learning objectives [41].

Although we have many years of experience in facilitating games, few of us had any formal pedagogical background when we began our careers as game facilitators. None of us could be classified as a professional facilitator. Even if we facilitated regularly, it was never the primary task of our work obligations. Only MK and SM had game facilitation and GBL as a formal part of previous education at the master’s level, and a few of us (SM, MK, and JBH) had aspects of GBL as a part of their PhD studies.

We were expected to be familiar with competency models—either explicitly or implicitly; information collected was from a general, teaching, and facilitation perspective. We asked ourselves whether we ever felt the need for a formal competency model during our game facilitation activities.

Our overall knowledge about competency models was inhomogeneous; 3 of us (AML, JBH, TB) neither knew of any models nor missed their absence, while 4 (HS, SM, TL, MK) had a general understanding or awareness of competency models. Various competency models were listed, but all were more relevant for higher education and vocational training [42-45]. One author (AML) pointed out that most studies on facilitation only cover small groups (fewer than 100 participants). We did not specifically feel that a formal competency model was lacking. Except for 2 (MK and JBH) people, we never felt a need for a formal competency model. All agreed that a formal competency model might be useful when changing and adapting guidelines for game facilitation. Two authors commented that facilitation of highly customized and user-specific games is not supported by existing competency models. The collected data indicate that the roles of the facilitator change depending on whether the facilitator handles the whole teaching unit or only manages the game facilitation.

Regarding which competencies the authors felt were required, 2 further questions were asked: (1) Which competencies are important for the successful use of games from your point of view? (2) For which competencies do you see the biggest discrepancies between requirements and actual competencies? Where is there a need for training?

Most of us explained that we had little pedagogical knowledge when we started facilitating games (and teaching in general). The general consensus was to have the following competencies: “active listening”, as with “reactive “and “proactive abilities” to act on group reactions using strategies like “team management,” “participation techniques,” “consensus techniques,” “community management empathy,” “conflict resolution,” and “flexibility”; “ability to assess pure facilitation techniques” and to “integrate experiential learning principles” of “moderation,” “mentoring,” and “instructional capabilities under GBL settings”; and an “understanding of the toolsets” that can be implemented.

These led us to identify the following challenges: digital skills, leadership capabilities, cognitive science, and motivational skills.

Another major issue which was discussed was the preference for the facilitator to ask closed questions. In higher education it is important to have learners synthesize and create new knowledge. Many students lack this ability; however, the games are often designed for this purpose. Facilitators need to have skills to encourage perspective taking and reasoning to foster this process. Regarding the facilitation of large groups, there are many digital tools that can support such facilitation. One hypothesis, based on our reflection in discussions, is that increased digital competencies among teachers and students could ease the use of these tools. As this has to be considered as a relevant topic, more information on tools and methods can be found in Multimedia Appendix 1.

Our collective experience as authors is, as stated earlier, in engineering and management in higher education, mostly at the university level. This is limited when analyzing the overall field of facilitation in higher education. Hence, additional data were collected from a wider audience through structured interviews and an online survey.

### External Experience Review

This section analyses the experiences of 4 interviewees. The results were compared to the results of the author group (using the same questions) and the survey outcome. The 4 participants teach at different faculties, but all within engineering. Their experience in game facilitation ranged between 3 to over 10 years. All had been heavily involved in the development process of least one game. This differed from the author group, whose members had been additionally involved in the development of commercial off-the-shelf games. The topics the interviewees taught were urban mobility planning, traffic simulation, health care logistics, and games for ideation and innovation.

One facilitator used the same game twice in a course. The game was integrated into an existing curriculum and used at the beginning and at the end of the course in a workshop setting with undergraduate and graduate students, so that the students could experience the learning progress throughout the course. Another facilitator used the same game throughout the semester with graduate engineering students, deployed through a blended learning environment. The curriculum was based on (and constructed around) the game scenarios. The third facilitator had used the game in around 25 sessions. It was embedded in a course, similar to the experience of the first facilitator and was played in one room with a physical and a digital component. The fourth facilitator used a game in a workshop setting (half a day) for undergraduate students in civil engineering. It was a blended learning concept (as in the second case), comprising briefing, playing, and lecture. In all 4 cases, the usage of games was initiated because of research activities and all chairs having extensive experience in GBL. All 4 facilitators had at least 2 years of experience in facilitating games. They agreed that games can be motivating and deliver a different way of teaching. One emphasized the interactive and active learning activities in GBL.

Regarding the perspective on facilitation, the 2 participants with more extensive experience in facilitating explained more and paid more attention to the introductory setting than the participants with less experience. One interviewee reported that the game can lead to frustration, but also to high engagement. However, overall, interviewee knowledge about competency models was limited: 2 participants professed to having no knowledge of the models, 1 had a general understanding, and 1 knew about the different competency models but not related to teaching. None had ever used a competency model for facilitation. However, 3 stated to have overlooked or at least missed it. Before gathering their own experience with game facilitation, they would have liked to be more aware of the following: connection between game design and facilitation process; how to observe and what to observe; how to assess and conclude the game process; how to know what aspects or knowledge needs to be assessed; how to understand the players’ game decisions, “soft data’” as behavior of players, the level of communication with others etc; and how to observe learners in relation to providing feedback.

The final interview questions focused on the competencies the interviewees felt were required and on the gap. All interviewees mentioned the importance of knowing the subject, the tools used, and the technical environment as a required competency. One pointed out that it is imperative to also understand the methods behind GBL and that connecting gameplay and the intended learning outcome is a key success factor. Two of the interviewees mentioned the need for motivational competency. Three saw the necessity for a facilitator to integrate the game in a larger context from a didactical perspective. One interviewee mentioned the ability to respect and regard the competency of the students.

The answers concerning the gap focused on the lack of formal pedagogical courses on game didactics and how to integrate games into the curriculum. The interviewees also saw a deficiency in competency for facilitating groups of extreme sizes (ie, fewer than 4 or over 100 students) and how to deal with a group’s inhomogeneity in relation to the game runtime and different levels of knowledge. Further, 1 interviewee mentioned a dearth of methods for nurturing the reflection competency among the students during the game session and in the debriefing phase.

The results of the qualitatively analysis indicated a large uniformity related to facilitation and on the perception of required competencies. In order to obtain quantifiable data and a broader data source, an online survey was designed.

### Questionnaire

This section describes the development of the questionnaire and its results. The questionnaire supplemented and refined the qualitative data with quantitative data on facilitation and competencies. The questionnaire was drafted by JBH, HS, and TB. It was followed by a pretest and a subsequent discussion among all authors to validate it. The questionnaire comprised 5 parts: (1) demographic data, (2) general questions on game facilitation, (3) questions on the most challenging game facilitation, (4) a section on the importance of a competency model, and (5) personal training received. The questionnaire concluded with an open question for comment on any other important topic.

The questionnaire was distributed via social media in the authors' personal networks and professional communities between June 1, 2020, and June 13, 2020, with 30 participants taking part in the survey. In the remainder of this section, the results specifically relevant for facilitating are described. Further results are included in Multimedia Appendix 2.

#### Demographics

The participants’ teaching experience in higher education ranged from 2-30 years, with an average of 12.5 years (SD 7.02). There was an average of 9.3 years of game facilitation experience (SD 6.73; range 1-30). In addition, the participants stated that they had facilitated an average of 12.2 (SD 20.32) games. Here, the span was wide, ranging from 1 to 100, with 1 value (999) being considered an outlier. As most participants had facilitated more than one game, this percentage differs from the percentage of educational scenarios. In terms of positions, 37% (11/30) of participants, the largest group, were professors and senior lecturers, 23% (7/30) were senior academics, 23% (7/30) were other academics, 13% (4/30) were PhD students, and the remaining 3% (1/30) included other positions.

When asked about the rationale for the use of games (with multiple selections being permitted), 83.% (25/30) of participants indicated their own personal initiative, 67% (20/30) responded that a game was the best fit for the intended learning outcomes, 57% (17/30) used games to contribute to multifaceted teaching methods, 30% (9/30) of the participants stated that the game was available at the respective institution, and only 13.3% (4/30) of the participants used games because games were part of the curriculum. Overall, it can be stated that the games in higher education are not systematically anchored and are rather used because of the personal initiative of the lecturers.

**Figure 1:**
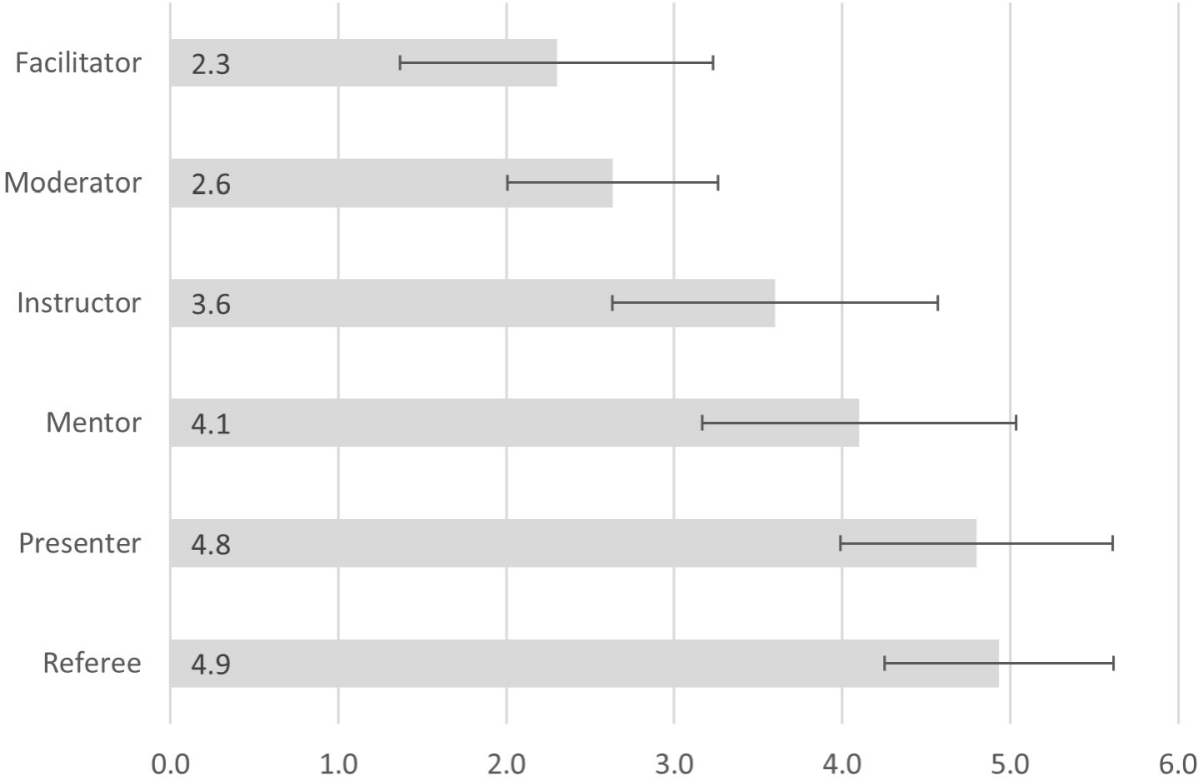
Ranked roles in game facilitating (7-point Likert scale; N=30).

Game facilitation comprises various roles. Thus, the participants were asked to rank 6 predefined roles taken during facilitation. For determining the statistical parameters, nonranked roles were given the value 7. Figure 1 shows that the role of the facilitator (average rank 2.3) and the role of the moderator (average rank 2.6) were considered the most important. The role of the instructor, which was ranked third (average rank 3.6), was followed by the mentor role (average rank 4.1). The presenter role (average rank 4.8) and the referee role (average rank 4.9) occupied the last 2 ranks. Overall, the results show a coherent picture of the importance of the different roles of a game facilitator. Table 1 shows the distribution of game types facilitated by participants.

**Table 1:** Game types facilitated (multiple selections per respondent; N=30).

Game type	Frequency, n (%)
Specially developed serious games	22 (73)
Analogue games (eg, board games)	18 (60)
Analogue simulation games	14 (47)
Blended serious games (ie online and offline and /or digital and analogue)	14 (47)
Off-the-shelf games	13 (43)
Commercial entertainment games	12 (40)
“Modded” commercial entertainment games (ie, technically adapted)	6 (20)
Other	3 (10)

#### Challenges in Game Facilitation

**Figure 2:**
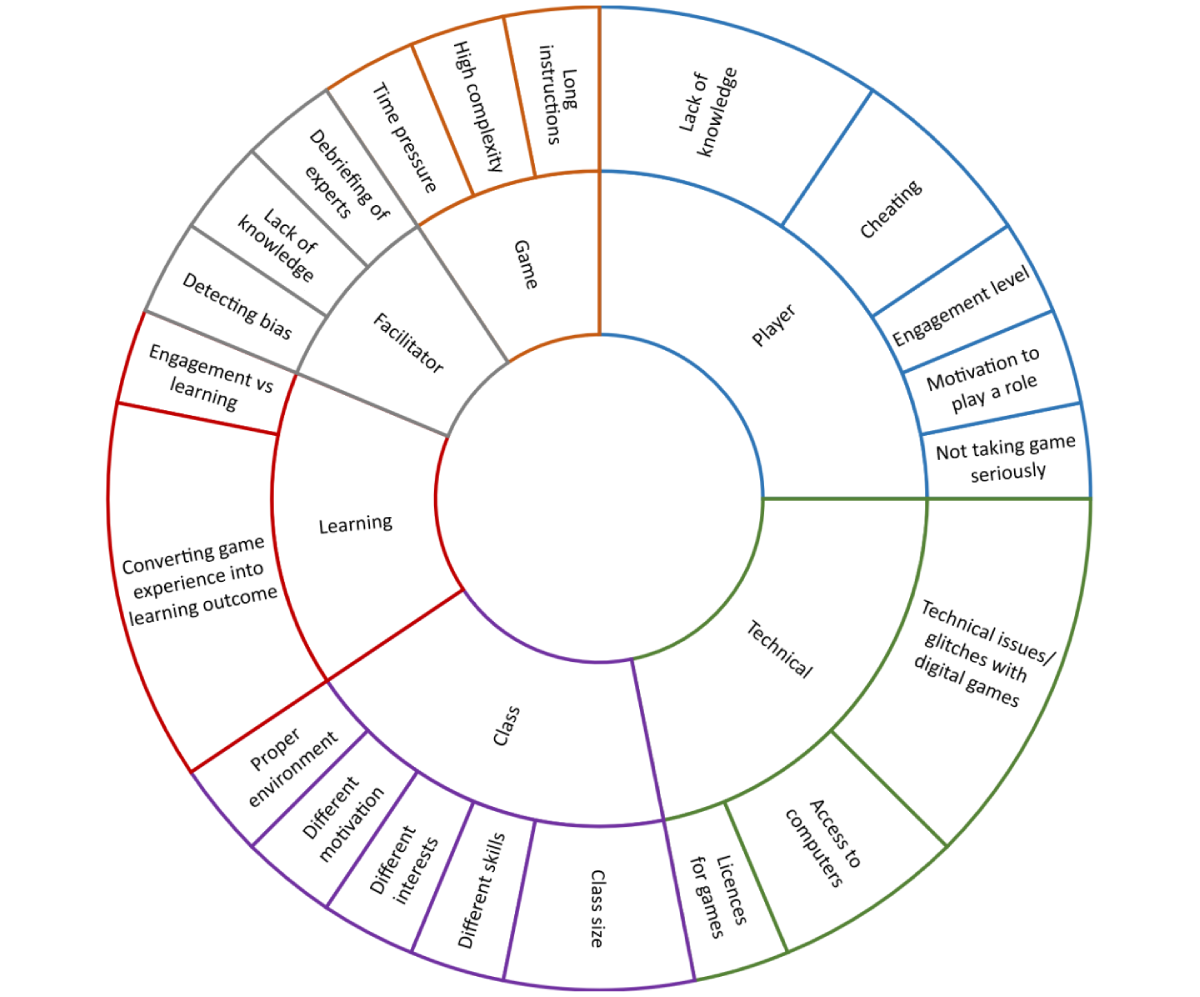
Cluster diagram of challenges in game facilitation as elicited by open-ended questions (N=18).

With an open-ended question, the participants were requested to describe their most challenging game facilitation and to specify the challenges they faced. Using theme analysis, we identified 32 challenges from 18 of the answers. With each challenge being unique, they were clustered into groups based on common themes and shared characteristics. For example, some participants mentioned that players do not have sufficient knowledge or that players cheat. Although these examples are different, they still describe the characteristics or behavior of players. Hence, to ease the understanding of all challenges in game facilitation, they were grouped into 6 clusters: challenges related to (1) individual players and their actions, (2) technical aspects of using games, (3) class and collective aspects of players, (4) learning aspects of games, (5) games themselves, and (6) facilitators. Figure 2 shows these groups and challenges. The inner circle shows the clustered challenges and the outer circle the challenges themselves.

Challenges related to players include lack of knowledge to make all required decisions in a game. An example here might be a game for urban planning where students lack knowledge associated to economic aspects related to planning decisions. Furthermore, some students may not consider a game as a serious pedagogical tool. Convincing students to engage in game activities is challenging even without the added difficulty of students not fulfilling their role in the game.

Some challenges are technical in the context of digital games. Issues with software, device compatibility, or bugs or glitches in the game itself can be highly disruptive. Another challenge is student accessibility to computers or the internet to participate in a game.

Cohort sizes cause additional challenges on implementing games for learning, as discussed in the External Experience Review subsection and in Multimedia Appendix 1. A further issue is maintaining motivation for all players by ensuring the right environment during the entire game process. Mixed groups, in terms of skills, interests, and motivation can also be a challenge to the game facilitator.

As the purpose of games for learning is to ensure certain learning outcomes, converting the experiences of playing a game into these outcomes remains another major challenge. A concern mentioned by participants is that many students do not recognize the link between games and the subject of the overall program. Therefore, finding a proper balance between engagement and learning is a necessity. Immersion is important, but too high a level may hinder the learning effects.

Difficulties related to gameplay have implications for learning. The learning curve with respect to the game’s usage and interaction can add additional complexity into a game learning experience. However, overly simple games may be boring and insufficient to achieving learning outcomes, while overcomplicated games may be too hard, not engaging, and may distract from intended learning outcomes.

Finally, some challenges are related to the facilitators themselves. Situations where a facilitator does not detect cognitive biases in individual and collaborative activities would consequently lead to an incorrect assessment of the game experience. Facilitating a game where experts are present can be a challenge too, because of the demand to define the problem and solutions. The latter challenges relate directly to game facilitation and reflect the competencies required of game facilitators. The challenges in the other categories in Figure 1 were reported by game facilitators to be of lower priority for game facilitation (Table 2). Many of the challenges may be not relevant during the facilitation itself, but become more pertinent when preparing the GBL activity and the infrastructure required. Thus, further expertise might be necessary, including, for example, when managing technical problems, such as licenses or access to computers, or when grappling with game-related challenges, such as high complexity or long instructions. Overall, the challenges outlined above suggest that successful use of GBL depends on good game facilitation, but also on many other aspects. The following section examines what training game facilitators receive to master these challenges.

**Table 2:** Prioritization of competencies (5-point Likert scale; N=30).

Competency		Stewart^a^ (mean)	Study (mean)	Difference
**Interpersonal competencies (communication skills)**				
	Verbal	4.9	4.5	–0.4
	Nonverbal	4.6	3.4	–1.2
	Written	4.2	3.2	–1.0
	Questioning	4.8	4.2	–0.6
	Active listening	4.8	4.2	–0.6
	Perceptive listening	4.6	4.1	–0.5
	Empathy	4.3	4.1	–0.2
	Summarizing/paraphrasing	4.6	3.9	–0.7
	Sensitivity to group	4.6	4.3	–0.3
**Interpersonal competencies (further skills)**				
	Sensitivity to underlying emotions	4.5	3.9	–0.6
	Cultural awareness	4.5	3.8	–0.7
	Encouragement of participation	4.4	4.3	–0.1
	Negotiation skills	4.5	3.5	–1.0
	Flexibility	4.8	4.3	–0.5
	Conflict recognition	4.5	4.0	–0.5
	Conflict resolution	4.3	3.7	–0.6
	Conflict transformation	4.2	3.6	–0.6
	Leadership	4.1	3.7	–0.4
	*Motivating others to achieve goals* ^b^	*4.0*	*4.1*	*0.1*
	*Motivating others to participate creatively*	*4.2*	*4.4*	*0.2*
	*Recognizing/rewarding achievement*	*3.5*	*4.1*	*0.6*
	Model neutrality	4.6	3.8	–0.8
	Building relationships	4.3	3.9	–0.4
**Management process competencies**				
	*Planning/organizing*	*4.4*	*4.5*	*0.1*
	Managing time	4.5	4.3	–0.2
	Managing audiovisual aids	4.4	3.5	–0.9
	Managing physical environment	4.4	3.4	–1.0
	Assimilating information	4.1	3.8	–0.3
	*Coaching others*	*3.8*	*3.9*	*0.1*
	Managing feedback	4.4	4.3	–0.1
	Managing contract	4.1	3.2	–0.9
**Understanding context competencies**				
	Understanding organizational context	4.4	4.0	–0.4
	Knowledge of theory and application of group facilitation	4.1	3.9	–0.2
**Personal characteristics**				
	Adaptability	4.7	4.6	–0.1
	Intellectual agility	4.5	4.3	–0.2
	Trustworthiness	4.6	4.1	–0.5
	Results motivation	4.3	3.7	–0.6
	Objectivity	4.5	3.9	–0.6
	Emotional resilience	4.7	3.9	–0.8
	Self-awareness	4.6	3.9	–0.7
	Self-development	4.3	3.8	–0.5

^a^Values in this column are from the paper by Stewart [33].

^b^Italics indicate competencies which scored higher for game facilitation over group facilitation.

#### Facilitation Training

The participants surveyed were also asked about the facilitation training they received (Table 3): 87% (26/30) selected “Learning by doing”, 57% (17/30) selected “Co-facilitating with colleagues”, and 47% (14/30) selected “Work shadowing with colleagues”. It is worth noting that 2 of the 3 responses (“Learning by doing” and “Co-facilitation with colleagues”) cannot be considered structured training and only a third of the participants received formal training (“Formal course in university pedagogy”). “Supervision by experienced colleagues” was mentioned by only a quarter of the participants, with supplier-specific training accounting for the remaining portion of replies. Overall, the proportion of those with formal training was rather low. This low rate of formal training may reflect the overall low rate of structured pedagogical training in university teaching.

**Figure 3:**
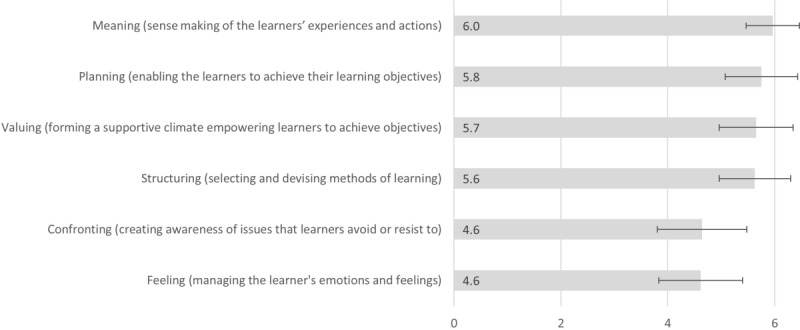
Helpfulness of trainings for categories according to Heron [46] (7-point Likert scale; N=27).

The taxonomy of Heron [46] was used to determine the extent to which training was helpful. Six dimensions of facilitation were defined in this taxonomy. On a 7-point Likert scale, participants were asked to rate for each dimension the degree to which the training received supported the respective dimension of facilitation (Figure 3). At the top of the scale with a value of 6.0 points was the dimension “Meaning”, on which, as evidenced by the low SD, the participants largely agreed. The other dimensions with similarly high scores but larger SDs were “Planning” (mean score 5.8), “Valuing” (mean score 5.7), and “Structuring” (mean score 5.6). The dimensions “Confronting” and “Valuing” were rated on average 1 point (mean score 4.6) lower.

In summary, the high scores for all dimensions indicate that training was generally perceived as helpful. It remains to be clarified whether this assessment is because of the comparatively low amount of formal training, or whether the quality of facilitation might be strengthened by further targeted training.

**Table 3:** Types of training received (multiple selections per respondent; N=30).

Type of training	Frequency, n (%)
Learning by doing	26 (87)
Cofacilitating with colleagues	17 (57)
Work shadowing with colleagues	14 (47)
Formal course in university pedagogy	10 (33)
Supervision by experienced colleagues	8 (27)
Training course at the supplier or third party institution	7 (23)
None	2 (7)
Other	1 (3)

#### Competency Model

Stewart’s [33] competency model for group facilitators comprises 41 competencies categorized in 5 groups. The competence model was selected because group facilitation is usually included in game facilitation. Thus, Stewart’s model represents a valid initial foundation for further tailoring to game facilitation processes, as similarly concluded by Kortmann and Peters [38]. In this study, the participants were asked to rate the importance of each of the competencies for game facilitators on 5-point Likert scales (Table 2). Almost without exception, game facilitator competencies received lower scores for the importance of each competency.

Table 4 lists all competency group scores of the participants surveyed. The difference of 0.7 points for the group of communication skills as the most important competency group for group facilitation, is striking. For game facilitation covered by this study, all scores are in the narrow range of 3.9 to 4.0 and below the scores for group facilitation investigated by Stewart [33]. Potentially, games being in the focus of GBL activities act as compensating media, which require less competence from game facilitators than is necessary for group facilitators.

On average, the competencies of Stewart's model [33] received for game facilitation scored 0.5 points lower compared to group facilitation. To determine which competencies were important for game facilitation compared to group facilitation, only competencies which scored higher for game facilitation over group facilitation were selected (Table 2, italics). The most significant competence was recognizing/rewarding achievement“ with a difference of 0.6, indicating a typical characteristic of games: recognition and rewards. In second with a difference of 0.2 was motivating others to participate creatively, which is associated with games where motivation plays a prominent role. Competencies with a difference of 0.1 included motivating others to achieve goals. Finally, coaching others and planning/organizing were considered by the respondents more important for game facilitation over group facilitation.

**Table 4:** Difference of prioritization compared to Stewart [33] per competency group.

Competency group	Stewart^a^ (mean)	Study (mean)	Difference
Interpersonal competencies (communication skills)	4.6	3.9	–0.7
Interpersonal competencies (further skills)	4.2	3.9	–0.2
Management process competencies	4.3	3.9	–0.4
Understanding context competencies	4.3	4.0	–0.3
Personal characteristics	4.5	4.0	–0.5

^a^Values in this column are from the paper by Stewart [33].

## Discussion

### Study Outline

Over the past decades, there has been a shift from teacher-centric toward learner-centric learning models [47], which has also nurtured the uptake of experiential learning, including GBL. This shift has also affected the teacher’s role, but it remains unclear how this change has influenced the teacher’s competencies regarding facilitation within higher education. Teachers within higher education often lack the formal pedagogical education that teachers within the K12 system have. In this study, therefore, the following 3 research questions were investigated: (1) Which competencies are particularly needed by the facilitator, and what are the training needs of the facilitator? (2) What do the relevant training courses for occasional game facilitators in higher education look like? (3) How do the competencies of occasional game facilitators differ from the competencies needed for other learning approaches, such as lectures, problem-based Learning, or online education?

### Which Competencies Are Particularly Needed by the Facilitator, and What Are the Training Needs of the Facilitator?

Core competencies according to the interviews and authors’ reflection were divided into 2 major groups: managerial and technical competencies. The more technical competencies comprise topics such as the knowledge of the gameplay, game content, its connection to the intended learning outcome, and the operation of any technical infrastructure. The more managerial competencies include active listening and reactive and proactive abilities to act on groups reactions, through the use of strategies like team management, leadership, motivational and participation techniques, consensus techniques, community management empathy, conflict resolution, and flexibility. This is underpinned by the survey (Table 2), which prioritized the competencies for game facilitation designated by Stewart [33]: verbal communication received a score 4.5 (out of 5), motivating others to participate creatively scored 4.4, and flexibility and encouraging participation (all interpersonal competencies) both scored 4.3. The personal characteristics adaptability and intellectual agility scored 4.6 and 4.3, respectively. Competencies like planning and organizing (score 4.5), and managing time and feedback (both scores 4.3) were also seen as core competencies, which is in line with the qualitative results.

The second part of this research asked about the need for training. The answer to this was complicated by the low proportion of people who received training (formal and informal). Among the interviewees, none had formal pedagogical training. This holds for two-thirds (67%, 20/30; Table 2) of the survey group, while we seem to have a higher percentage of formal course completion (10/30, 33.3%; Table 2). This might be because more than half of us hold faculty positions with requirements on pedagogical training for higher education. The qualitative results clearly state the need for training in the connection of the pedagogy and gameplay from a different perspective.

The actual training in the area of game facilitation for all was low, which can be seen also in the following wish list of the interviewees on the addressed training topics before first facilitation: connection between game design and facilitation process (Heron: meaning); how to observe and what to observe (Heron: meaning); how to assess and conclude the game process; how to know what aspects of knowledge need to be assessed (Heron: planning and structuring); how to holistically understand players’ game decisions and “soft data” as representations of player behavior, and the level of communication with others (Heron: confronting and feeling); and how to observe players for feedback purposes.

According to Figure 3, training in general was perceived as helpful. Looking at the wish list, it becomes apparent that a training program focusing on facilitation would support the formal competency development and also fill a need identified by many of the respondents.

### What Do the Relevant Training Courses for Occasional Game Facilitators in Higher Education Look Like?

The answer to this question was somewhat negative (Table 2). In the survey, 87% (26/30) indicated that their training consisted of learning by doing. In the author group, fewer than 20% (1/7) had received formal training before starting their game facilitation. Overall, the share of formal training remains low. Compared with K12 teachers or professional vocational trainers, this low rate of formal training may reflect the overall low rate of structured pedagogical training in university teaching. There is, however, a change in higher education. Although, scientific excellence used to play an overarching role in applying for academic faculty positions, there has been a shift toward also focusing on teaching experience and competency over the past decade. This may relate also to an increased focus on the process quality within higher education [48]. More and more countries have imposed formal requirements on pedagogical competencies, as a part of the appraisal procedure. Maturity models are widely used for assessing the maturity level within a specific area [49,50]. Despite not being frequently used, different maturity models also exist for higher education. For example, Zhou [51] has developed a capability maturity model of the e-learning process. Game facilitation is not primary about e-learning, as many games are haptic or board games, but Zhou includes the dimension of process capability in his model, which is transferable for game facilitation and higher education. It comprises the following levels (those transferrable from e-learning to GBL): delivery, delivers facilitated GBL units; planned, outlines clear and measurable objectives for GBL projects; defined, provides a defined process for development and support of GBL; managed, ensures the quality of the resources and the deliveries; and optimizing, continually improves in all aspects of the process. In matching the outcomes of the received training focusing on facilitation, it can be concluded that maturity is maximum at level 1of the model proposed by Zhou [51]. This might also be a reason why the uptake is so low. Staff interested in using games for education must undergo a time-consuming process of learning by doing. This leads to difficulties in delivering an acceptable quality of teaching during the first years occupying this role. When we consider the fact that most facilitators only use these methods once or twice a year, it is easy to see why there might be a problem.

As there is arguably hardly any training for facilitation of games provided and an increasing number of universities are offering programs on didactics in higher education, it would be valuable to know how the participants in this study rate the differences in competency needs.

### How Do the Competencies Of Occasional Game Facilitators Differ From The Competencies Needed for Other Learning Approaches, Such as Lectures, Problem-Based Learning, or Online Education?

Table 2 and Table 4 illustrate this issue. With reference to Stewart’s competency model, it can clearly be stated that the participants see large differences in quite a few areas. For communication skills, the overall tendency is that this is less important in the facilitation of games, where nonverbal and written communication showed the highest difference (1.2 and 1.0, respectively), but is nonetheless still relevant. This might be seen in the light of the answer given to the question of what type of games the participants use: a large majority indicated that they use games with in-built communication. For interpersonal skills, there are also large deviations, specifically on negotiation (difference of 1.0); however, these skills might also considered part of the gameplay, and thus not so relevant for the facilitation. According to Table 2, the differences for competencies on motivation related to goal achievements, creative participation, and reward and recognition are quite low; however, there is a higher need for these competencies. These are also competencies that were identified on the wish list in the qualitative part of the study. On the other hand, personal characteristic competencies seem to be less needed.

### Summary and Future Work

It can be concluded that the maturity of game facilitation in higher education is low. There is a need for formal training courses, with competency models rarely being implemented in this field. Besides implementing training for game facilitators, further approaches are available for increasing the diffusion of serious games and the effectiveness of GBL. Figure 2 provides an overview of the challenges involved—such as motivating players—to be covered by approaches for increasing the diffusion of serious games. For example, giving learners a choice to take part in GBL activities or to engage in some other learning activities is likely to increase learner motivation in the chosen learning activity [15,52]. Likewise, the choice of the game used itself has a great impact on learning success: learners have preferences for games depending on learner traits, such as age, ethnicity, and gender [53,54], but certain game mechanics might be especially suited for GBL activities [55,56].

This multimethod study investigated the competencies essential for game facilitation in higher education and analyzed, with use of empirical data, the perceived gap between essential and existing competencies. This paper also discusses if there is a structured approach for competency development for the target group. The findings indicate that there is a limited or no general competence model for game facilitators and that casual game facilitators rarely undergo any specific, formal training. The lack of specific training is to be regarded as one reason for the lack of dissemination of games in higher education. The study provides the basis for a competence model for game facilitators that may serve as a prerequisite for the development of specific trainings. Future work includes the confirmation, consolidation, and refinement of the competence model presented, for example, by means of an extended survey for a larger group of participants. Based on the competence model defined, we plan to develop organizational policies for training. With an increased dissemination of GBL provided by the growing of game facilitation competencies, the effects on teaching in higher education should be explored. However, one approach that could replace the training of game facilitators is the digital support or even the replacement of game facilitators by virtual assistants as supported by improvements in artificial intelligence [57].

## Appendix

Multimedia Appendix 1 Facilitation with large groups.

Multimedia Appendix 2 Further results from the questionnaire: educational scenarios.

## Abbreviations

GBL: game-based learning
