# Supplementary material for: Current Competencies of Game Facilitators and Their Potential Optimization in Higher Education: Multimethod Study
Source: JMIR Serious Games. 2021 May 5;9(2):e25481. doi: 10.2196/25481 (PMC8135020; doi:10.2196/25481)
Supplement: Multimedia Appendix 1 [file games_v9i2e25481_app1.pdf]

## Multimedia Appendix A: Facilitation with large groups

Designing facilitation activities for large audiences has to deal with an important scale factor to be taken into account. Executive education in business schools and corporate universities provide good examples that can be used in higher education at all levels. The use of social media, web technology and of collaborative platforms has been successfully implemented and already documented [1–4] in particular for what concerns the support to the debriefing phase of a GBL experience. Post-Game Learning Communities (PGLC) have been created to consolidate the game learning experience over time: the cohorts of participants (usually around 30 people, but up to hundred sometimes) have been gathered together around a community platform where they have had the opportunity to implement the learning outcomes that were devised by the game experience [5]. Here, the facilitator sets the floor to a learning-by-doing experience as a natural follow-up of the game experience and as long as that participants get confident with the new environment and the tasks they are given, plays more and more the role of an observer and a reporter of the activities performed in the platform in order to measure the learning impact on the audience that is, learners' ability to apply what they have been supposed to learn during the game experience.

These kinds of platforms [5] have been successfully replicated for different serious games in executive education and were also filling a gap in terms of awareness, knowledge and adoption of web 2.0 technologies among managers. Such experience has paved the way to the use of participative technologies and social media nowadays to support not only the debriefing phase and the follow-up of a game session, but the whole learning experience, game run included. Scalability is no longer a technical issue as video conferencing, social media, MOOCs, online collaboration tools and real-time online questionnaires are well established. The two last examples are significant as they provide solutions for blended learning settings and allow to get feedback from large gatherings in the real world. So, a facilitator has plenty of tools to tap in to reach large audiences. Therefore, the process of facilitation is more important than the instruments. The latter depends on the former. The process can be supported by the introduction of Liberating Structures [6] and cognate tools. These are more than a collection of facilitation techniques to foster innovation. They allow to address in a distributed and inclusive way complex situations by tapping into collective intelligence, provided that the proper settings are prepared. They have been designed to involve all participants, but the space arrangement plays an important role regardless of being physical, virtual or blended, give room for exchanges and self-expression. Here lies the secret to deal with facilitation for large audiences offline, online, and in blended environments: the more the facilitation is shifted towards delegation and self-regulation, the more effective and the better impact is expected. Large audiences are difficult to be controlled and observed at once. Therefore, the good strategy is to design facilitation in such a way to engage the audience in compelling activities involving everyone in first person [7] and with a shared purpose, by stimulating everyone's intrinsic motivation [8, 9] at the same time.

Official and unofficial Liberating Structures [10] can also be combined in sequences (strings), nested in one another as building blocks, and changed at will in as many variants as needed. Liberating Structures are conceived to a) share ideas, knowledge, experiences, b) reveal/discover opportunities/ generate ideas, c) analyze/diagnose/debrief, d) help/get help, e) strategize, f) plan. Even though they have been originally conceived for offline activities, they can be easily implemented online:

- "Gallery Walk" (aka "Shift and share") and "World Café" that best fit to spread knowledge and stimulate individual reflection on and team discussion around specific topics can be organized in breakout rooms.
- "Mini Constellations", "Critical Uncertainties", "Agreement-Certainty Matrix" and "Ecocycle Planning" are addressing issues and challenges visually on a map to express respectively the level of agreement, the different perspectives, and phases, all can be implemented by the combination of a videoconferencing system and a whiteboard as co-editing support – the facilitator has just to explain the context and the rules;
- "Wise crowds" (aka "Co-development" or "Mastermind") and "Fishbowl" which focus on peer support and experience exchanges (particularly useful for experiential learning implemented via role-playing games) can be implemented by a video conferencing tool with or without the use of breakout rooms – the facilitator communicates the context and the rules, moderates the requests of intervention/involvement in the ongoing discussion and distributes teams in breakout rooms if required.

For what concerns brainstorming, the simplest way of implementing them on a large scale and asynchronously is using the so-called brainwriting technique.

The technical means and the procedural techniques are available to tackle any kind of audience offline and online, nowadays. What is most important with large groups is to change the facilitator's mindset that has to be keener on adopting a designer attitude in preparing the settings of the intervention and in involving the audience in active and self-regulating participation by leveraging on collective intelligence and inclusive facilitating techniques.

## References


1. Angehrn, A.A.: Experience with Post-Game Learning Communities (PGLC), https://seriousgamessociety.org/2016/09/22/experience-with-post-game-learning-communities-pglc/.
2. Catalano, C.E., Luccini, A.M., Mortara, M.: Guidelines for an effective design of serious games. Int. J. Serious Games. 1, (2014).
3. Luccini, A.M.: How to set up Post Game Learning Communities, ttps://seriousgamessociety.org/2016/09/22/how-to-set-up-post-game-learning-communities/.
4. Luccini, A.M.: Why to set up Post Game Learning Communities, https://seriousgamessociety.org/2016/09/22/why-to-set-up-post-game-learning-communities/.
5. Angehrn, A.A., Maxwell, K., Marco Luccini, A., Rajola, F.: Designing effective collaboration, learning and innovation systems for education professionals. Int. J. Knowl. Learn. 5, 193–206 (2009). https://doi.org/10.1504/IJKL.2009.031195.

6. Lipmanowicz, H., McCandless, K.: The surprising power of liberating structures: Simple rules to unleash a culture of innovation. Liberating Structures Press Seattle, WA (2013).
7. Axelrod, R.: Terms of engagement: New ways of leading and changing organizations. Berrett-Koehler Publishers (2010).
8. Deci, E.L., Flaste, R.: Why we do what we do: Understanding self-motivation. Penguins Books (1995).
9. Pink, D.H.: Drive: The surprising truth about what motivates us. Penguin (2011).
10. Group Jazz: Group Jazz handbook: Engaging Everyone with Liberating Structures.
