# Supplementary material for: Current Competencies of Game Facilitators and Their Potential Optimization in Higher Education: Multimethod Study
Source: JMIR Serious Games. 2021 May 5;9(2):e25481. doi: 10.2196/25481 (PMC8135020; doi:10.2196/25481)
Supplement: Multimedia Appendix 2 [file games_v9i2e25481_app2.pdf]

Multimedia Appendix B: Further Results from the Questionnaire - Educational Scenarios

The questionnaire included also questions aiming at the educational scenarios, i.e. in what form the game is embedded in the teaching. Again, the participants could choose from a multiple selection question (see **Error! Reference source not found.**). With 80.0% (24/30) the most common option is embedding the game in a lecture. The game is used in one lecture in such a scenario to achieve certain learning goals, allowing the preceding and following lectures to be designed independently of the game. The second most frequently mentioned scenario is the homework variant with 46,7% (14/30): learners are given the homework assignment to work with a game to achieve certain goals. Often such a scenario is prepared and followed up (briefing, debriefing) in the pre- and post-lecture. 43.3% (13/30) of the participants use a game over several lectures while considering the same scenario. Here game facilitation accompanies the learners over a longer time and can also be described as a joint long-term developing of a particular scenario. The variant next frequently mentioned 36,7% (11/30) is similarly structured: here, game facilitation also extends over several lectures. However, usually with the same game, different goals are aimed at.

Table 1. Educational scenarios used in facilitation (multiple selections per respondent, N=30).

**Educational Scenario**	**Frequency**
Compact in one lecture	80.0% (24/30)
As homework	46,7% (14/30)
Spread over several consecutive lectures (continuous scenario)	43.3% (13/30)
Spread over several consecutive lectures (different scenarios)	36,7% (11/30)
Other	6.7% (2/30)

Cohort size is also characteristic of an educational scenario. The most frequently mentioned cohort size is 16 - 30 (43%), followed by 6 - 15 (37%). A cohort size of over 31 learners is mentioned by 17% of the participants, while cohorts of less than 6 learners are indicated by only 3% of the participants.

The organizational conditions of educational scenarios have been evaluated (Table 2). The three most frequently referred to conditions of educational scenarios, with around 80% of the participants naming each (see Table 2), are the communication between the learners, the utilization of multiplayer games and the use of one venue each. In 46.7% (14/30) of the entries the game instances are connected via a network, e.g. "the students played individual 'cities' that were connected to 5-6 other students' 'cities' in the virtual environment.". The use of single player games (36.7%, 11/30), players distributed across different locations (36.7%, 11/30), no communication between players (6.7%, 2/30) and multiplayer games without interaction between players (3.3%, 1/30)) are hardly mentioned. Interestingly, in 20.0% (6/30) of the

responses the community of the game used is included in the communication of the players beyond the educational scenario. Complicating conditions for some games are the need for software licenses (36.7%, 11/30) and the ties to dedicated rooms (23.3%, 7/30).

Table 2. Organizational conditions (multiple selections per respondent, N=30).

Organizational condition	Frequency
With communication	83.3% (25/30)
Multiplayer game in which the players interact with each other in teams	80.0% (24/30)
Players in one location	76.7% (23/30)
Networked game instances	46.7% (14/30)
Licenses necessary	36.7% (11/30)
Players distributed across multiple locations	36.7% (11/30)
Single player	36.7% (11/30)
Tied to a specific room by technical requirements	23.3% (7/30)
Multiplayer game in which the players interact with each other at community level	20.0% (6/30)
Other	6.7% (2/30)
Without communication	6.7% (2/30)
Multiplayer game in which there is no interaction between the players	3.3% (1/30)
